# Author Correction: Bio-inspired nitric-oxide-driven nanomotor

**DOI:** 10.1038/s41467-019-10437-0

**Published:** 2019-05-21

**Authors:** Mimi Wan, Huan Chen, Qi Wang, Qian Niu, Ping Xu, Yueqi Yu, Tianyu Zhu, Chun Mao, Jian Shen

**Affiliations:** National and Local Joint Engineering Research Center of Biomedical Functional Materials, School of Chemistry and Materials Science, Nanjing Normal University, 210023 Nanjing, China

**Keywords:** Nanoparticles, Biochemistry, Materials chemistry, Bioinspired materials, Nanoscale devices

Correction to: *Nature Communications* 10.1038/s41467-019-08670-8, Article published online 27 Feb 2019.

The original version of this Article contained an error in the first sentence of the second paragraph of the ‘Influence of HLA_10_ nanomotors on the cells (HUVECs and MCF-7)’ section of the Results, which incorrectly read ‘In order to verify the universality of the formation mechanism of HLA_n_ nanomotors proposed in this case, that is, the positively charged amino-enriched organic substances and the negative carboxyl groups in L-arginine combined through weak electrostatic force to form nanoparticles, three amino-enriched organic compounds (chitosan, (Mw. 5–100 million), polylysine (Mw. 3000–4000), heparin/folic acid (FA) (Mw. 5000–10,000)) were chosen to react with L-arginine, and the morphology/movement behavior of the obtained nanoparticles (named as CLA_10_ nanomotors, PLA_10_ nanomotors, HFLA_10_ nanomotors) were investigated (Fig. 7 and Supplementary Movie 7).’ The correct version states ‘chitosan, (Mw. 3000–6000 Da)’ in place of ‘chitosan, (Mw. 5–100 million)’. This has been corrected in both the PDF and HTML versions of the Article.

