# Supplementary material for: Bio-inspired nitric-oxide-driven nanomotor
Source: Nat Commun. 2019 Feb 27;10:966. doi: 10.1038/s41467-019-08670-8 (PMC6393443; doi:10.1038/s41467-019-08670-8)
Supplement: Supplementary file 1 — Supplementary Information [file 41467_2019_8670_MOESM1_ESM.docx]

**Supplementary information**

*Bio-inspired Nitric Oxide-driven Nanomotor*

Wan et al.


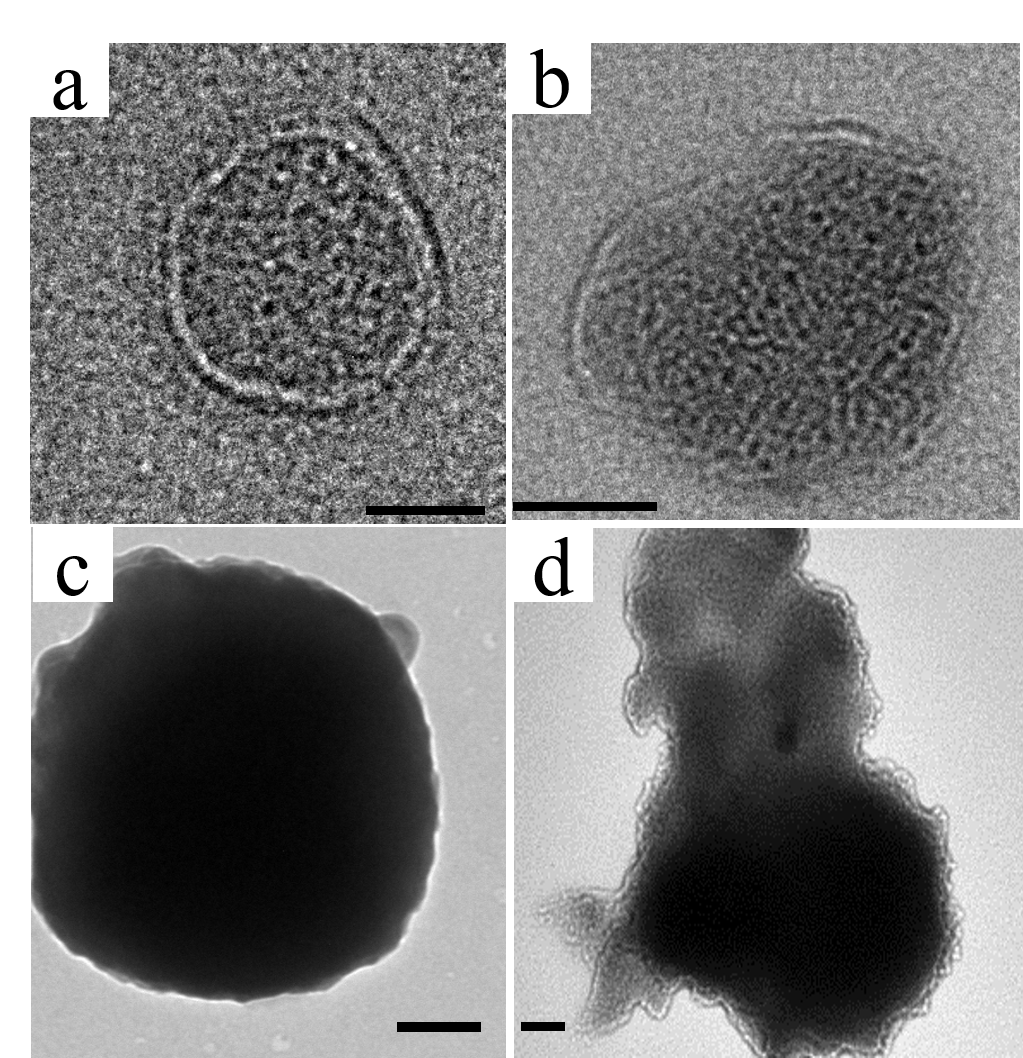


**Supplementary Figure 1.** Characterization of the samples. TEM images of (a) HLA_5_ nanomotor, (b) HLA_10_ nanomotor, (c) HLA_15_ nanomotor and (d) HLA_20_ nanomotor (Scale bar: 50 nm) .

**Supplementary Figure 2.** Particle size detected by dynamic light scattering (DLS) of HLA_5_, HLA_10_, and HLA_15_ nanomotors.

**Supplementary Figure 3.** The statistic analysis of the particle size. The statistics on particle size (obtained from TEM images, five distinct samples, 10 particles were taken from each image) for HLAn nanomotors.


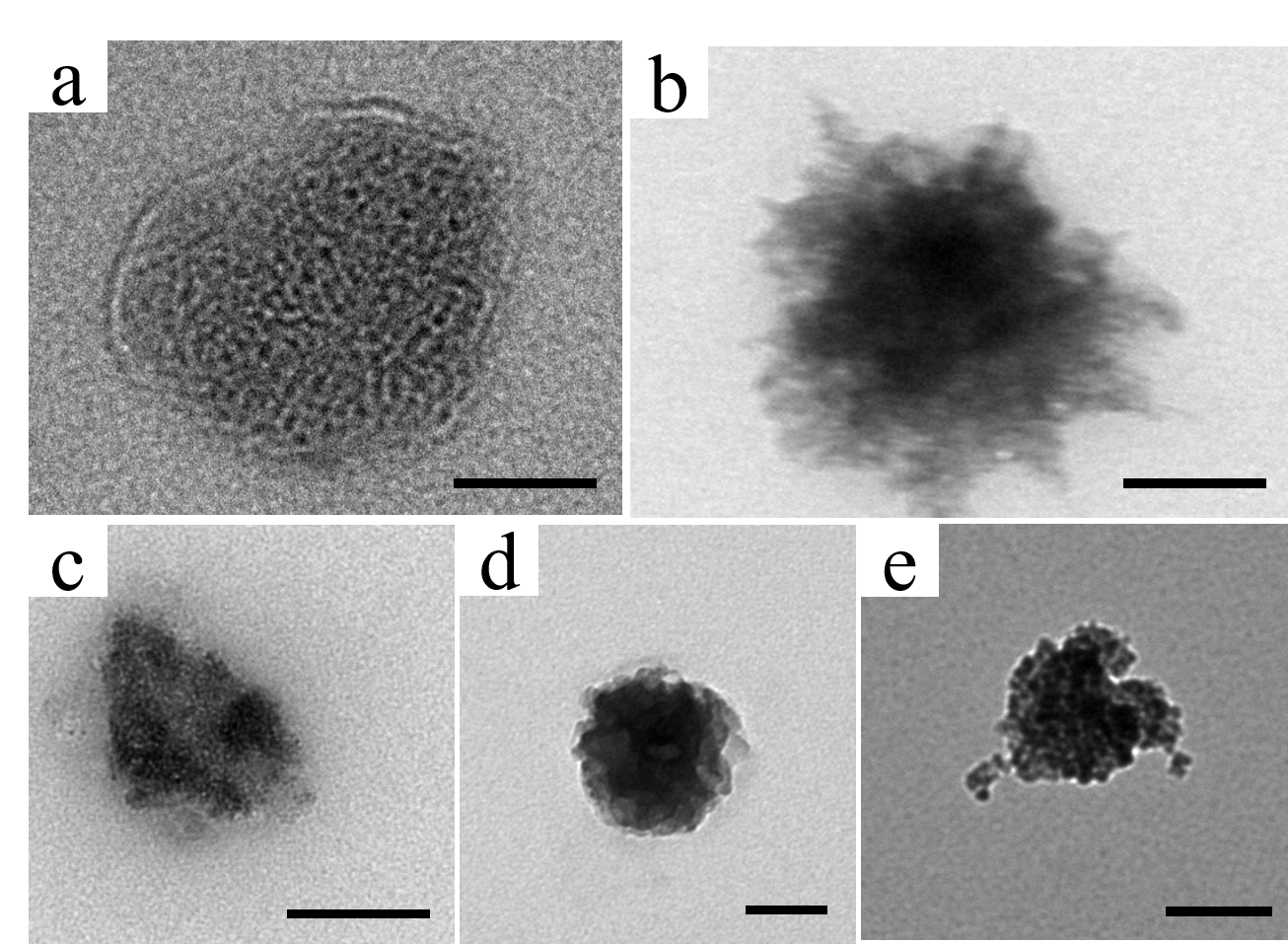


**Supplementary Figure 4.** Self-destroyed process of the nanomotors. Self-destroyed process of HLA_10_ nanomotor in 10% H_2_O_2_ for (a) 0 h, (b) 1 h, (c) 3 h, (d) 6 h, and (e) 18 h, respectively (Scale bar: 50 nm).

**Supplementary Figure 5.** Characterization of the samples. FTIR spectra of (a) HPAM, (b) HLA_10_ nanomotor, and (c) L-arginine.

**Supplementary Figure 6.** Characterization of the samples. FTIR spectra of (a) HLA_10_ nanomotor, and (b) HLC.


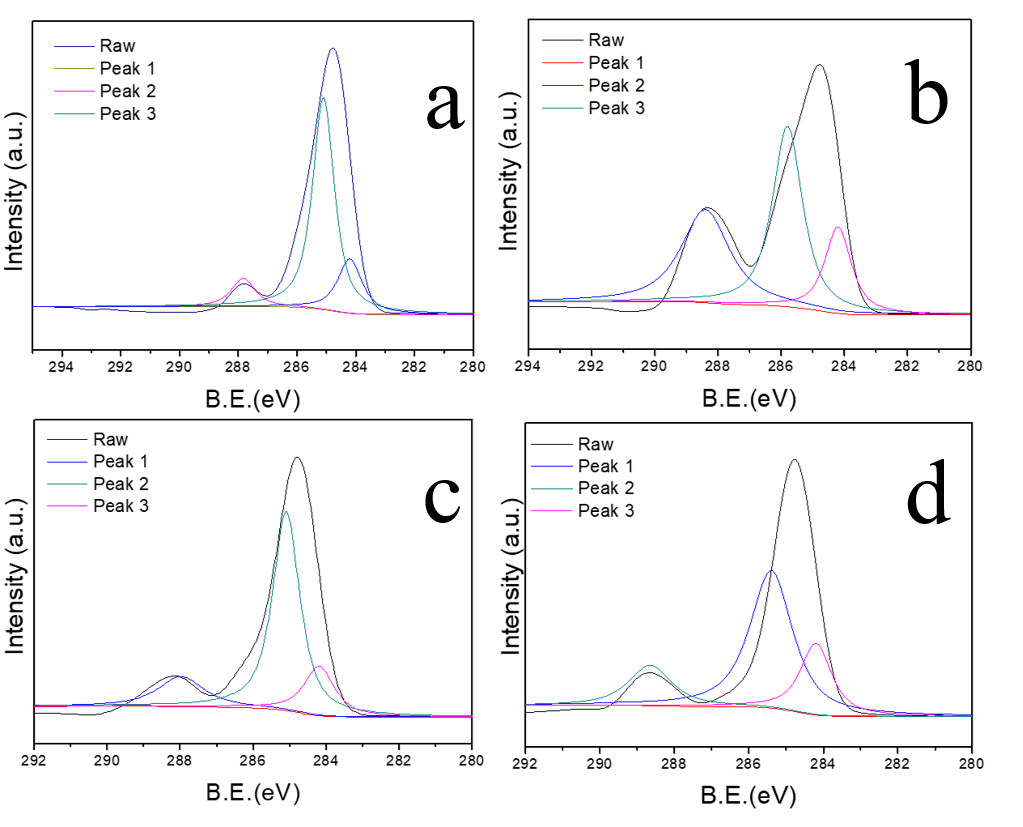


**Supplementary Figure 7.** Characterization of the samples. *XPS* (C1*s*) spectra of (a) HPAM, (b) L-arginine, (c) HLA_10_, and (d) HLC (supernatant HPAM/L-citrulline composite after HLA_10_ nanomotor reacting with 10% H_2_O_2_ for 24 h).

**Supplementary Figure 8.** Characterization of the samples. C1*s* spectra of (a) HPAM, (b) L-arginine, (c) HLA_10_, and (d) HLC (supernatant HPAM/L-citrulline composite after HLA_10_ nanomotor reacting with 10% H_2_O_2_ for 24 h).


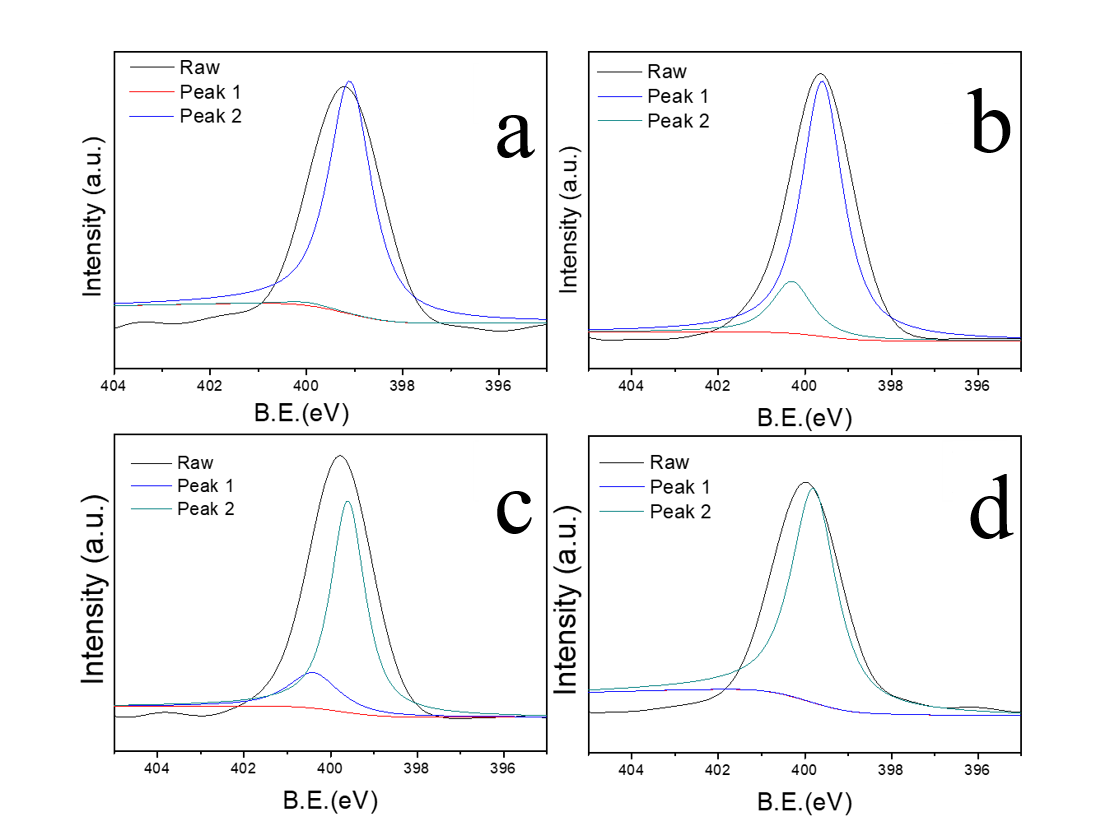


**Supplementary Figure 9.** Characterization of the samples. N1*s* spectra of (a) HPAM, (b) L-arginine, (c) HLA_10_, and (d) HLC (supernatant HPAM/L-citrulline composite after HLA_10_ nanomotor reacting with 10% H_2_O_2_ for 24 h).

**Supplementary Figure 10.** N1*s* spectra of (a) HPAM, (b) L-arginine, (c) HLA_10_, and (d) HLC (supernatant HPAM/L-citrulline composite after HLA_10_ nanomotor reacting with 10% H_2_O_2_ for 24 h).





**Supplementary Figure 11.** Chemical structure of FITC.

**Supplementary Figure 12**. Fluorescence property detection of the samples. Fluorescence spectra of different samples (Excitation wavelength: 490 nm).





**Supplementary Figure 13.** Chemical structure of heparin.

**Supplementary Figure 14.** Fluorescence property detection of the samples. Fluorescence spectra of (a) HPAM water solution (4 mg mL^-1^) with different excitation wavelengths, (b) HPAM water solution with different concentrations (excitation wavelength: 400 nm), and (c) HLA_n_ nanomotors.

**Supplementary Figure 15.** Hemolysis detection of the samples. Hemolysis of (a) HPAM, (b) L-arginine, and (c) HLA_10_ nanomotor. Experimental data points are mean + /- s.d. of triplicate samples in a representative experiment.


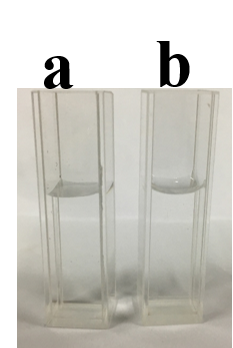


**Supplementary Figure 16**. Photographs of HLA_10_ dispersed in water (a) before and (b) after one-month storage.

**Supplementary Figure 17.** Particle size detection of the sample. Particle size detected by DLS of HLA_10_ dispersed in water (a) before and (b) after one-month storage (at room temperature).

**Supplementary Figure 18.** Particle size detection of the sample. Particle size detected by DLS of HLA_10_ prepared by five repeated times of experiment.


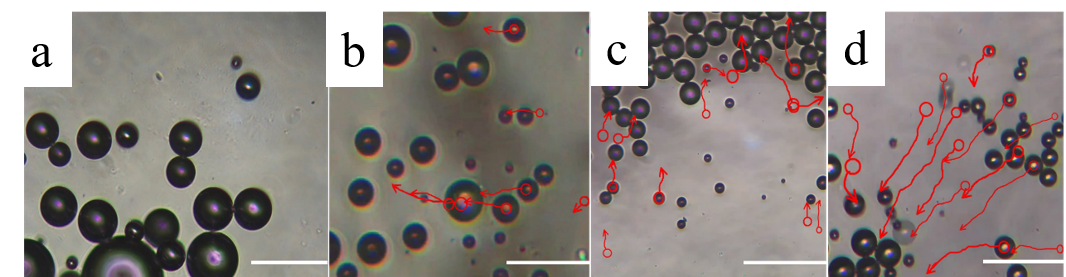


**Supplementary Figure 19.** Movement detection of the samples. Time-lapse images (Supplementary Movie 1) displaying the tracking trajectories of nanomotors under (a) HLA_5_, (b) HLA_10_, (c) HLA_15_, and (d) HLA_20_ in 15 s, respectively (Scale bar: 5 μm).


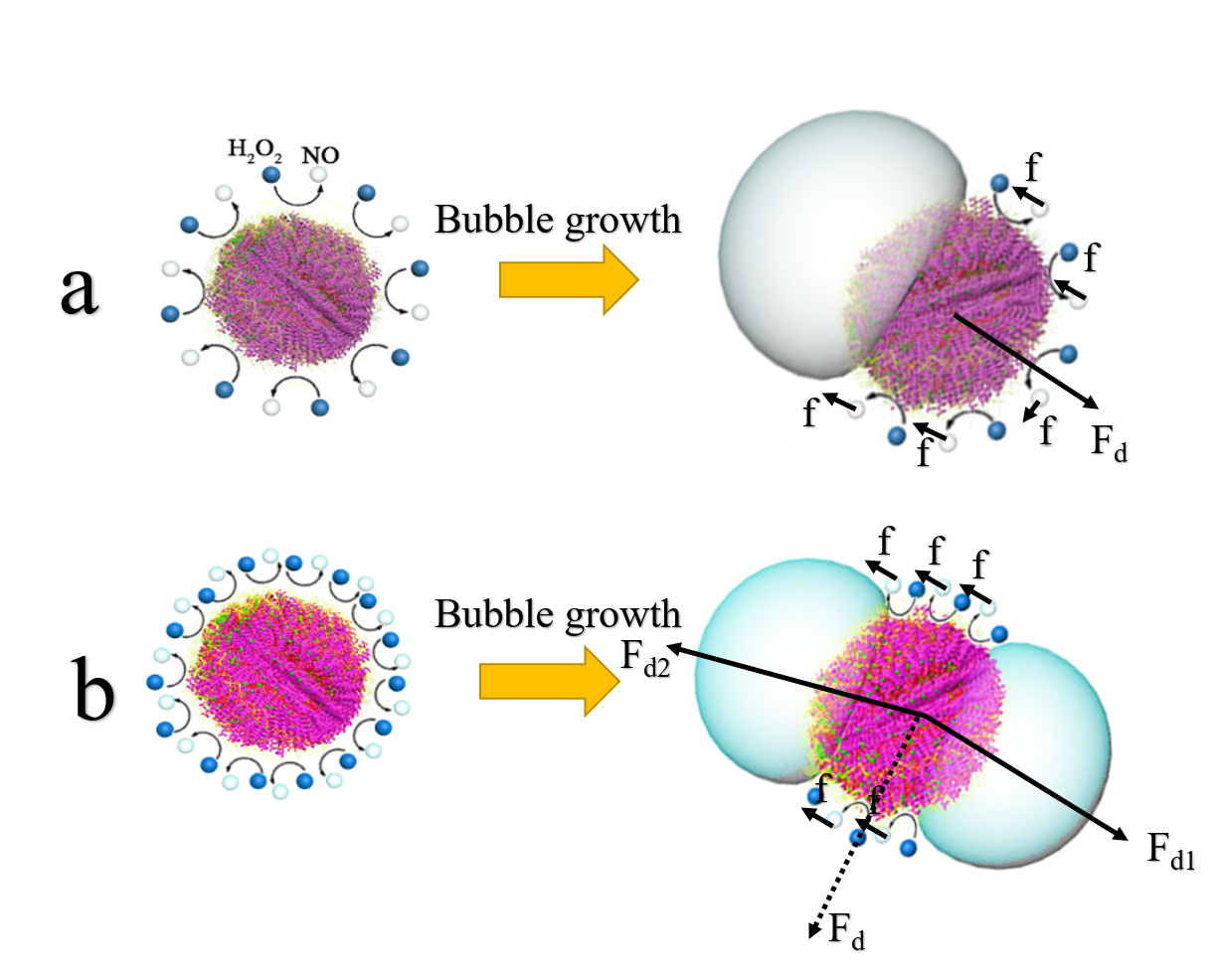


**Supplementary Figure 20.** Possible motion mechanisms of (a) the HLA_10_ and (b) HLA_15_/HLA_20_ nanomotors during bubble growth process.


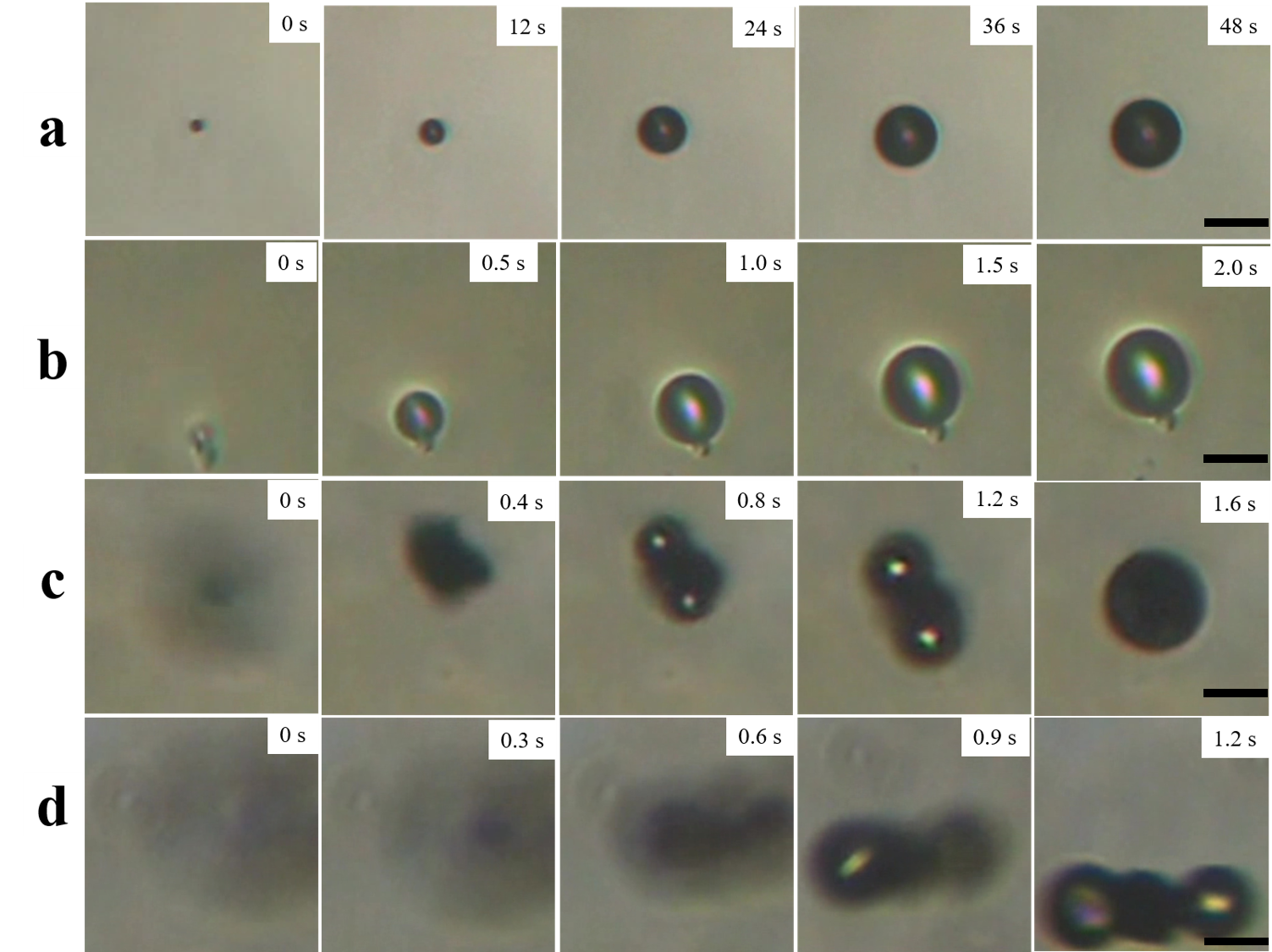


**Supplementary Figure 21.** Bubble formation process of the samples. Time-lapse images (Supplementary Movie 2) displaying the bubble generation process of (a) HLA_5_, (b) HLA_10_, (c) HLA_15_, (d) HLA_20_ in 10 s, respectively (20% H_2_O_2_) (Scale bar: 5 μm) .

**Supplementary Figure 22.** The statistic analysis of the speed. The statistics on speed for HLAn nanomotors (10 distinct samples, 5 nanomotors were taken from each Movie).

**
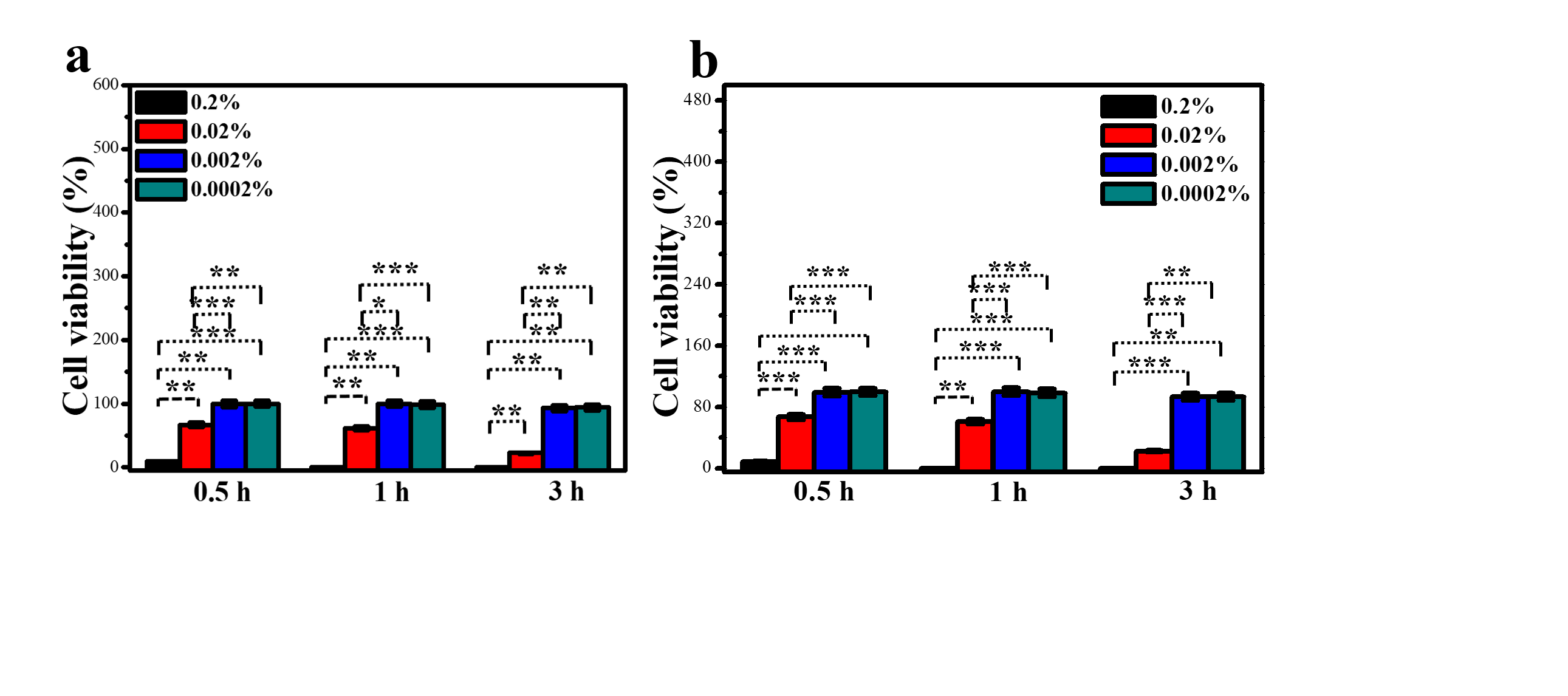
**

**Supplementary Figure 23.** Cell viability test. Cell viabilities of (a) MCF-7 and (b) HUVECs co-cultured with H_2_O_2_ under different concentrations. *Denotes statistical significance between bars (*for p < 0.05, ** for p < 0.01, *** for p <0.001) using one-way ANOVA analysis. Experimental data points are mean + /- s.d. of triplicate samples in a representative experiment.


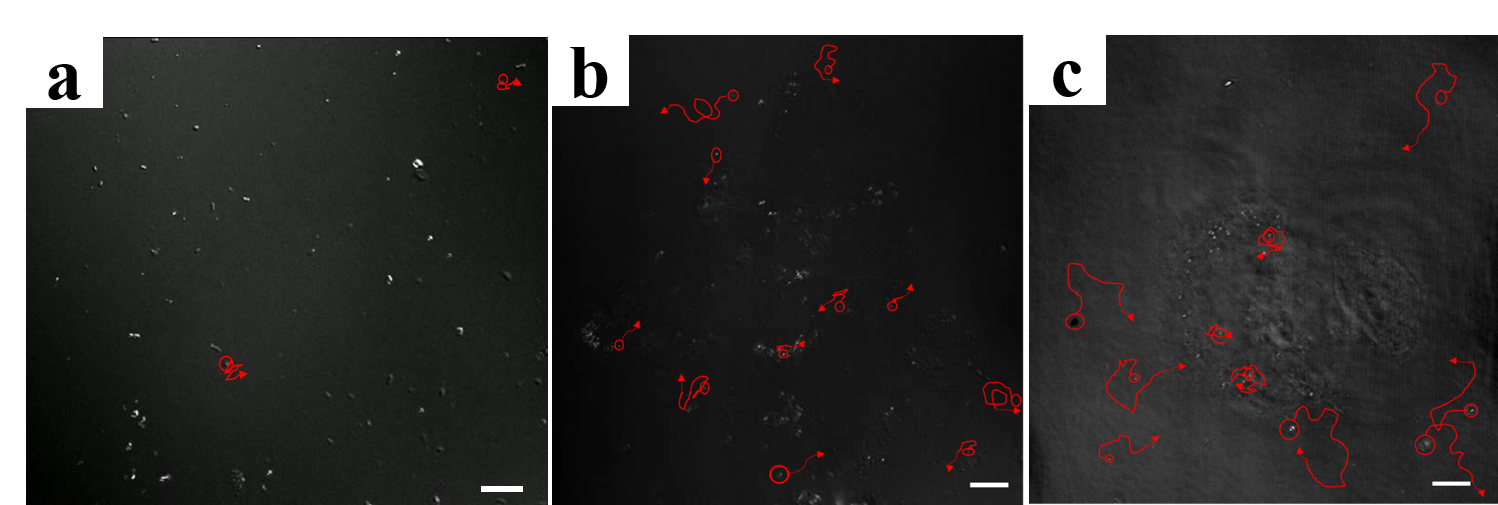


**Supplementary Figure 24.** Movement behavior of the nanomotors under cellular condition. Time-lapse images (Supplementary Movie 4) displaying the tracking trajectories of HLA_10_ nanomotor under (a) PBS solution, (b) MCF-7 cell solution, and (c) MCF-7 cell + 0.002%H_2_O_2_ in 1 s captured by confocal laser scanning microscopy (Scale bar: 5 μm).


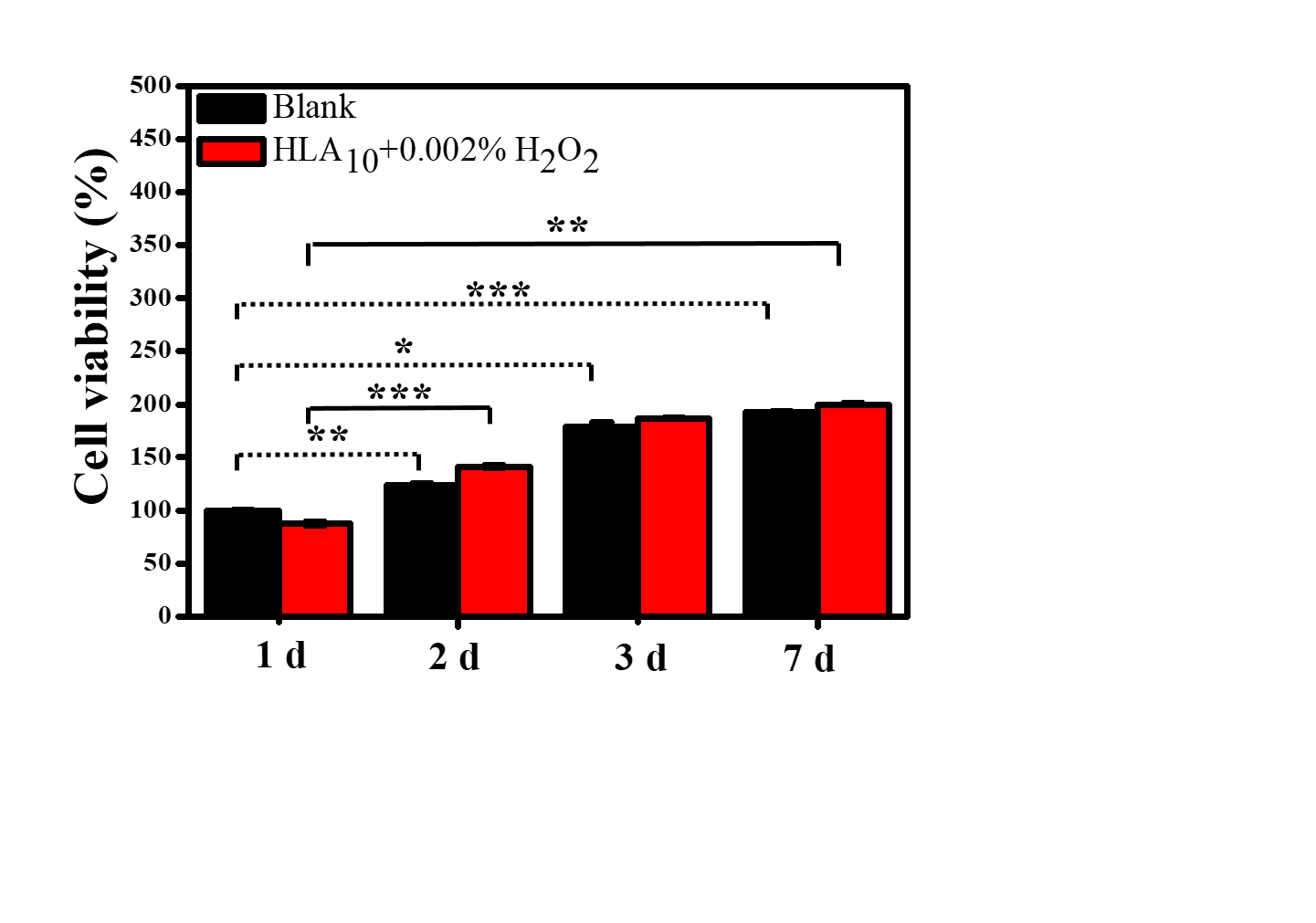


**Supplementary Figure 25.** Cell viability test. MTT results of HUVECs after cell-uptake process with HLA_10_ nanomotors under 0.002% H_2_O_2_ for different times. *Denotes statistical significance between bars (*for p < 0.05, ** for p < 0.01, *** for p <0.001) using one-way ANOVA analysis. Experimental data points are mean + /- s.d. of triplicate samples in a representative experiment.


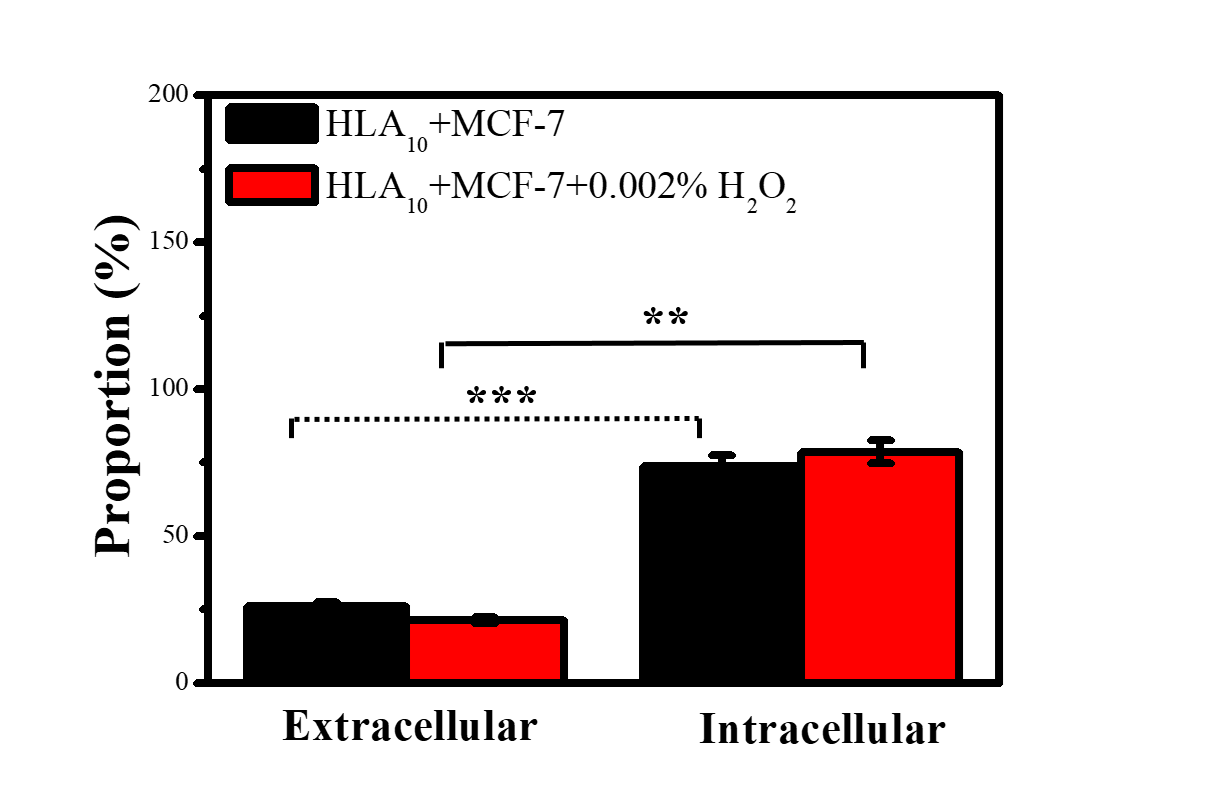


**Supplementary Figure 26**. NO production test. NO production proportion in intracellular and exreacellular condition by MCF-7 cultured with HLA_10_ nanomotor with and without 0.002% H_2_O_2_. *Denotes statistical significance between bars (*for p < 0.05, ** for p < 0.01, *** for p <0.001) using one-way ANOVA analysis. Experimental data points are mean + /- s.d. of triplicate samples in a representative experiment.


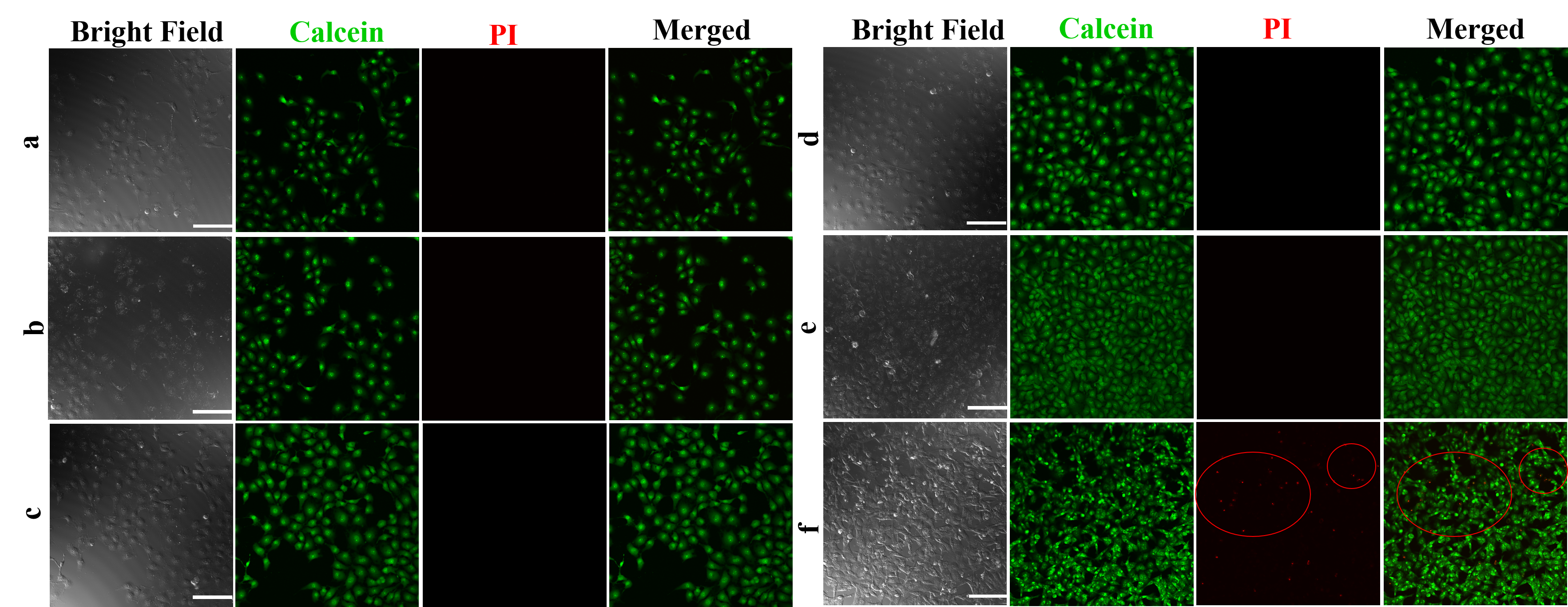


**Supplementary Figure 27.** Cell viability test. Live/Dead cell images of (a) HUVECs, (b) HUVECs+HPAM, (c) HUVECs+L-arginine, (d) HUVECs+HLA_10_ nanomotor 50 μL, (e) HUVECs+HLA_10_ nanomotor 100 μL, and (f) HUVECs+HLA_10_ nanomotor 150 μL(Scale bar： 100 μm) .


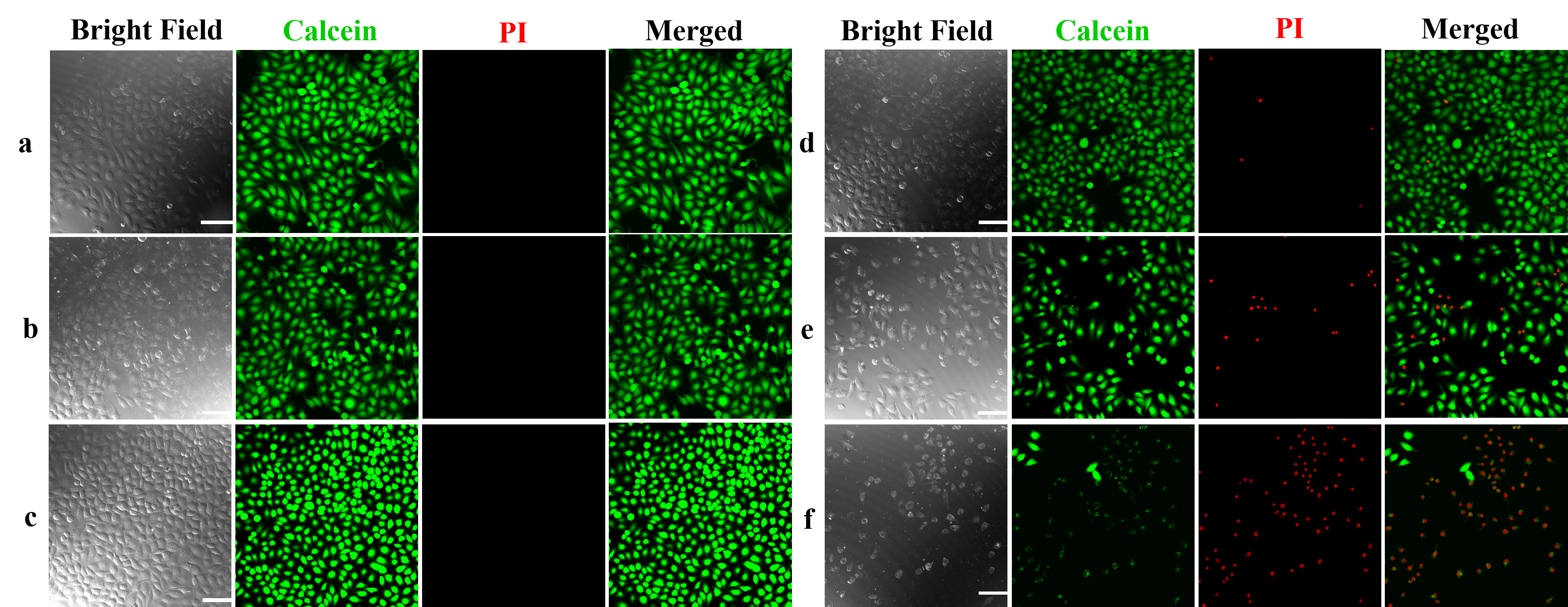


**Supplementary Figure 28.** Cell viability test. Live/Dead cell images of (a) MCF-7, (b) MCF-7+HPAM, (c) MCF-7+L-arginine, (d) MCF-7+HLA_10_ nanomotor 50 μL, (e) MCF-7+HLA_10_ nanomotor 100 μL, and (f) MCF-7+HLA_10_ nanomotor150 μL(Scale bar：100 μm).


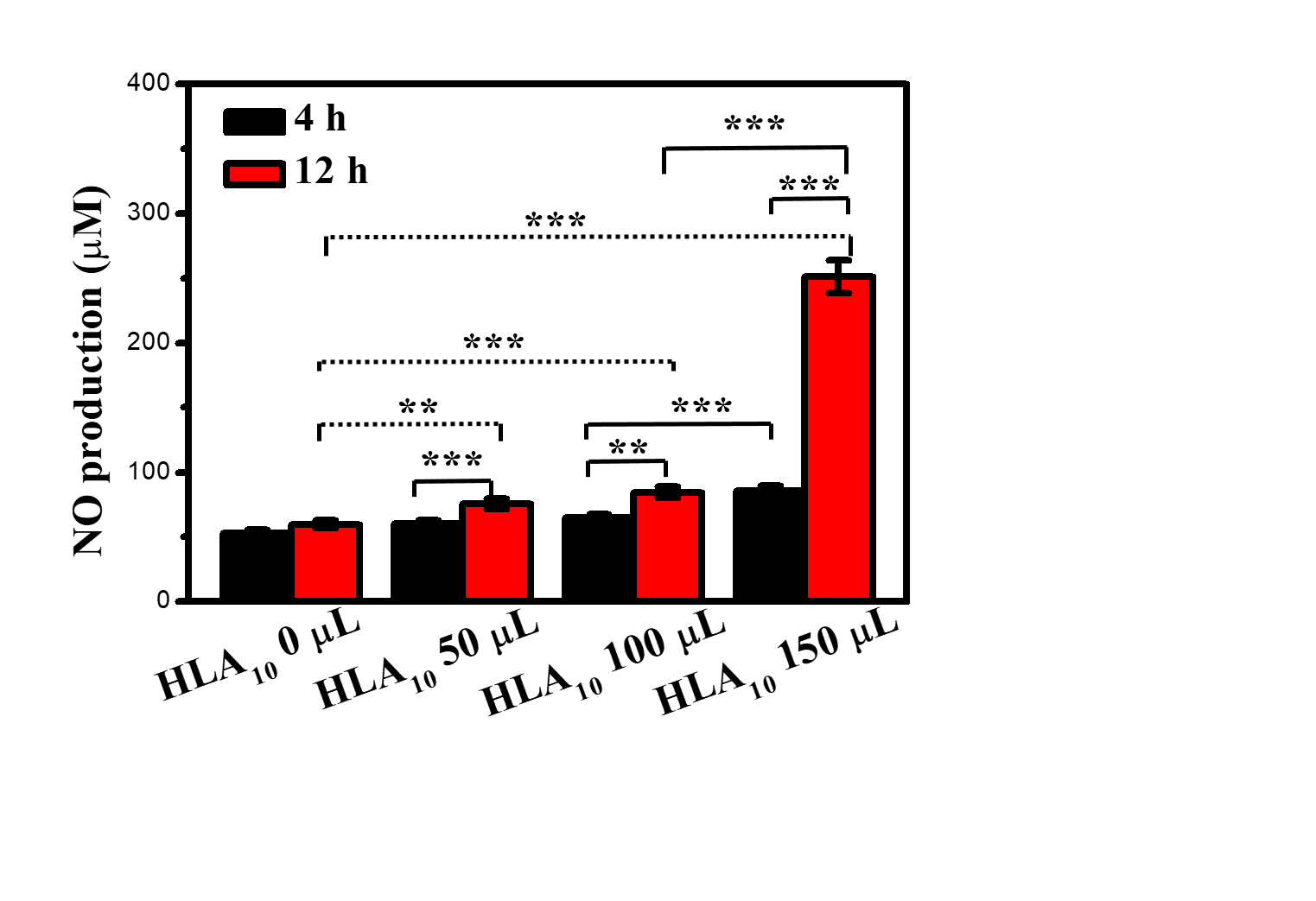


**Supplementary Figure 29.** NO production test. NO production amount during the co-culture of different amounts of HLA_10_ nanomotor with HUVECs for 4 h and 12 h, respectively. *Denotes statistical significance between bars (*for p < 0.05, ** for p < 0.01, *** for p <0.001) using one-way ANOVA analysis. Experimental data points are mean + /- s.d. of triplicate samples in a representative experiment.


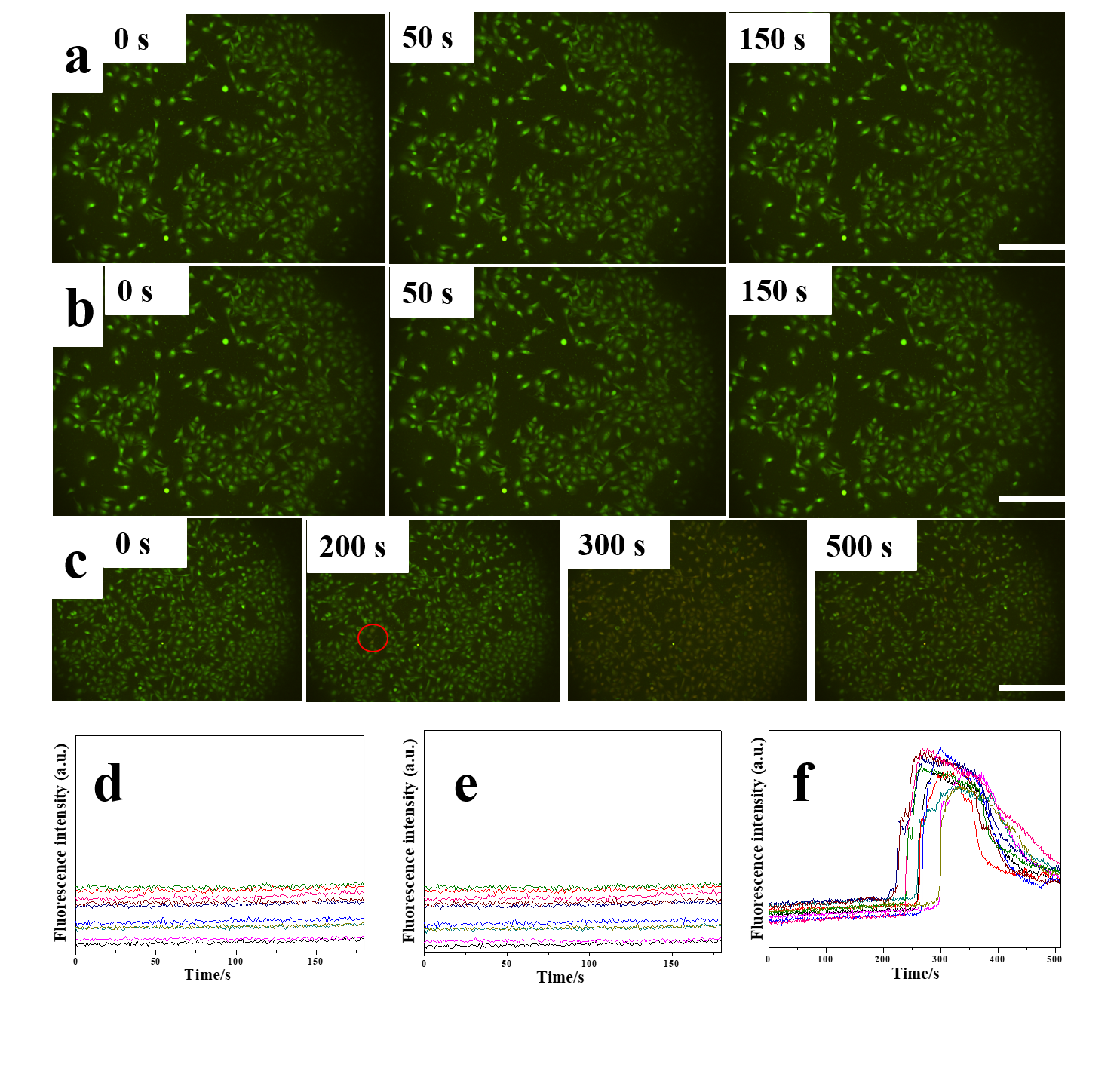


**Supplementary Figure 30.** Fluorescence Ca^2+^ imaging test. Fluorescence Ca^2+^ imaging of the cultured HUVECs after injection of (a) HPAM, (b) L-arginine, (c) HLA_10_ nanomotor, and the fluorescence intensity of the cells changes over time: (d) HPAM, (e) L-arginine, (f) HLA_10_ nanomotor (Scale bar: 50 μm) .


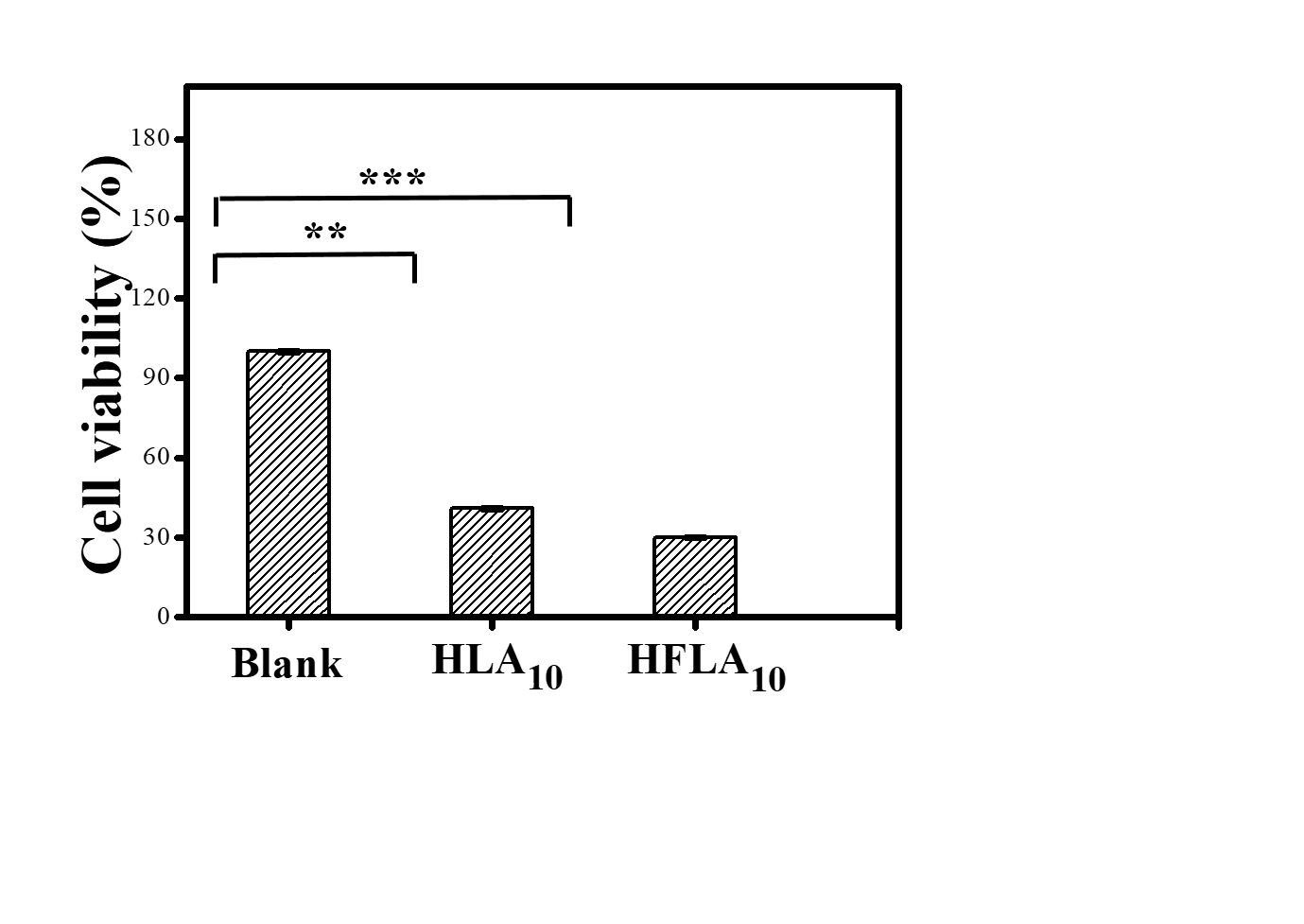


**Supplementary Figure 31.** Cell viability test. MTT results of different samples after co-culturing with MCF-7 for 3 h. *Denotes statistical significance between bars (*for p < 0.05, ** for p < 0.01, *** for p <0.001) using one-way ANOVA analysis. Experimental data points are mean + /- s.d. of triplicate samples in a representative experiment.

**Supplementary Figure 32.** Fluorescence property test. Fluorescence spectra of HLA_10_ and HFLA_10_.


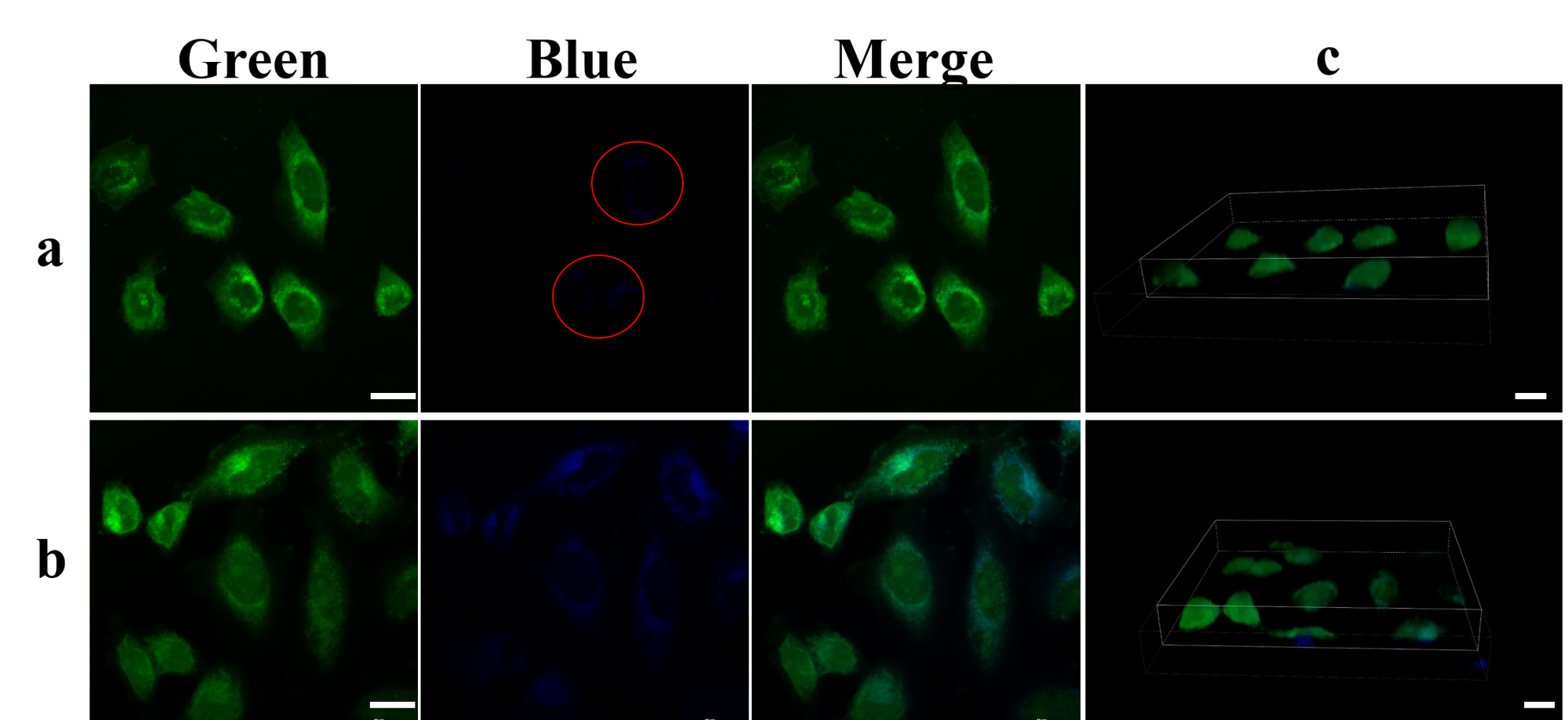


**Supplementary Figure 33.** Cellular uptake test. Confocal laser scanning microscopy images of the cellular uptake of (a) HLA_10_ and (b) HFLA_10_ nanomotors by MCF-7 cells and (c) a snapshot of a 3D rendered Movie (Supplementary Movie 8) made from a stack of confocal images. Blue: nanomotors; Green: cell membrane (Scale bar: 20 μm) .

**Supplementary Table 1.** A summary of reaction types explored for gas-nanomotor preparation

| Catalyst type | Fuel | Driving force | Reaction mechanism | By-products/  Undegradable substance | Reference |
| --- | --- | --- | --- | --- | --- |
| Pt | H_2_O_2_ | O_2_ | 2H_2_O_2_=2H_2_O+ O_2_↑ | Pt | 1-3 |
| Ag | H_2_O_2_ | O_2_ | 2H_2_O_2_=2H_2_O+ O_2_↑ | Ag | 4-6 |
| Fe | Acid | H_2_ | Fe+2H^+^=Fe^2+^+ H_2_↑ | Fe^2+^ | 7-9 |
| Ni | H_2_O_2_ | O_2_ | 2H_2_O_2_=2H_2_O+ O_2_↑ | Ni | 10-12 |
| Ru | H_2_O_2_ | O_2_ | 2H_2_O_2_=2H_2_O+ O_2_↑ | Ru | 13 |
| Al/Pd | Acid/base/ H_2_O_2_ | H_2_/ O_2_ | Al+3H_2_O=Al(OH)_3_+3H_2_↑  2H_2_O_2_=2H_2_O+ O_2_↑ | Al(OH)_3_ | 14 |
| MnO_x_ | H_2_O_2_ | O_2_ | 2H_2_O_2_=2H_2_O+ O_2_↑ | MnO_2_ | 15-17 |
| Mg | H_2_O | H_2_ | Mg+2H_2_O=Mg(OH)_2_+H_2_↑ | Mg(OH)_2_ | 18-20 |
| Zn | Acid | H_2_ | Zn+2H^+^=Zn^2+^+ H_2_↑ | Zn^2+^ | 21-23 |
| CaCO_3_ | Acid | CO_2_ | CaCO_3_+ 2H^+^=Ca^2+^+CO_2_↑ | Ca^2+^ | 246 |
| Urease | Urea | NH_3_/ CO_2_ | CO(NH_2_)_2_+H_2_O =2NH_3_↑+CO_2_↑ | Urease/NH_4_^+^ | 25 |
| TiO_2_ | H_2_O_2_ | O_2_ | 2H_2_O_2_=2H_2_O+ O_2_↑ | TiO_2_ | 26 |
| Glucose oxidase/ Catalase | Glucose | O_2_ | Glucose+ O_2_=Glucolactone+ H_2_O_2_/2H_2_O_2_= 2H_2_O+ O_2_↑ | Glucolactone | 27 |
| Catalase | H_2_O_2_ | O_2_ | 2H_2_O_2_=2H_2_O+ O_2_↑ | -- | 28 |
| No catalyst | Reactive oxygen | NO | L-arginine =L-citrulline+NO↑ | L-citrulline | This work |

**Supplementary References**

1. Sanchez, S., Ananth, A. N., Fomin, V. M., Viehrig, M. & Schmidt, O. G. Superfast motion of catalytic microjet engines at physiological temperature. *J. Am. Chem. Soc.* **133**, 14860-14863 (2011).
2. Gao, W., Sattayasamitsathit, S., Orozco, J. & Wang, J. Highly efficient catalytic microengines: template electrosynthesis of polyaniline/platinum microtubes. *J. Am. Chem.* *Soc.* **133**, 11862-11864 (2011).
3. Solovev, A. A., Mei, Y. F., Urena, E. B., Huang, G. S. & Schmidt, O. G. Catalytic microtubular jet engines self‐propelled by accumulated gas bubbles. *Small* **5**, 1688-1692 (2009).
4. Huang, C. X. & Shen, X. T. Janus molecularly imprinted polymer particles. *Chem. Commun.* **50**, 2646-2649 (2014).
5. Liu, M. et al. Micromotor based on polymer single crystals and nanoparticles: toward functional versatility. *Nanoscale*, **6**, 8601-8605 (2014).
6. Teo, W. Z., Wang, H. & Pumera, M. Beyond platinum: silver catalyst based bubble propelled tubular micromotors. *Chem. Commun.* **52**, 4333-4336 (2016).
7. Teo, W. Z., Zboril, R., Medrik, I. & Pumera, M. Fe^0^ nanomotors in ton quantities (10^20^ units) for environmental remediation. *Chem. Eur. J.*, **22**, 4789-4793 (2016).
8. Karshalev, E. et al. Utilizing Iron's Attractive Chemical and Magnetic Properties in Microrocket Design, Extended Motion, and Unique Performance. *Small*, **13**, 1700035 (2017).
9. Wang, Q. L., Wang, C., Dong, R. F., Pang, Q. Q. & Cai, Y. P. Steerable light-driven TiO_2_-Fe Janus micromotor. *Inorg. Chem. Commun.* **91**, 1-4 (2018).
10. Fournier-Bidoz, S., Arsenault, A. C., Manners, I. & Ozin, G. A. Synthetic self-propelled nanorotors. *Chem. Commun.* **4**, 441-443 (2005).
11. Ma, X. & Sanchez, S. A bio-catalytically driven Janus mesoporous silica cluster motor with magnetic guidance. *Chem. Commun.* **51**, 5467-5470 (2015).
12. Li, J., Xiao, Q., Jiang, J. Z., Chen, G. N. & Sun, J. J. Au-Fe/Ni alloy hybrid nanowire motors with dramatic speed. *RSC Adv.* **4**, 27522-27525 (2014).
13. Wang, W., Duan, W. T., Zhang, Z. X., Sun, M., Sen, A. & Mallouk, T. E. A tale of two forces: simultaneous chemical and acoustic propulsion of bimetallic micromotors. *Chem. Commun.* **51**, 1020-1023 (2015).
14. Gao, W., D’ Agostino, M., Garcia-Gradilla, V., Orozco, J. & Wang, J. Multi-fuel driven Janus micromotors. *Small* **9**, 467-471 (2013).
15. Chen, X. L., Wu, G., Lan, T. & Chen, W. Autonomous micromotor based on catalytically pneumatic behavior of balloon-like MnO_x_-Graphene crumples. *Chem. Commun.* **50**, 7157-7159 (2014).
16. Feng, X. M. et al. Graphene-based highly efficient micromotors. *Chem. Lett.* **44**, 399-401 (2015).
17. Singh, A. K., Mandal, T. K. & Bandyopadhyay, D. Magnetically guided chemical locomotion of self-propelling paperbots. *RSC Adv.*, **5**, 64444-64449 (2015).
18. Mou, F. Z., Chen, C. R., Ma, H. R., Yin, Y. X., Wu, Q. Z. & Guan, J. G. Self-propelled micromotors driven by the magnesium-water reaction and their hemolytic properties. *Angew. Chem. Int. Ed.* **52**, 7349-7353 (2013).
19. Li, J. X. et al. Water-driven micromotors for rapid photocatalytic degradation of biological and chemical warfare agents. *ACS Nano* **8**, 11118-11125 (2014).
20. Li, J. X. et al. Enteric micromotor can selectively position and spontaneously propel in the gastrointestinal tract. *ACS Nano* **10**, 9536-9542 (2016).
21. Gao, W. et al. Artificial micromotors in the mouse’s stomach: A step toward in vivo use of synthetic motors. *ACS Nano* **9**, 117-123 (2015).
22. Chen, C. R. et al. Transient micromotors that disappear when no longer needed. *ACS Nano* **10**, 10389-10396 (2016).
23. Gao, W., Uygun, A. & Wang, J. Hydrogen-bubble-propelled zinc-based microrockets in strongly acidic media. *J. Am. Chem. Soc.* **134**, 897-900 (2012).
24. Guix, M., Meyer, A. K., Koch, B. & Schmidt, O. G. Carbonate-based Janus micromotors moving in ultra-light acidic environment generated by HeLa cells in situ. *Sci. Rep.* **6**, 21701 (2016).
25. Ma, X., Wang, X., Hahn, K. & Sánchez, S. Motion control of urea-powered biocompatible hollow microcapsules. *ACS Nano* **10**, 3597-3605 (2016).
26. Dong, R. F., Zhang, Q. L., Gao, W., Pei, A. & Ren, B. Y. Highly efficient light-driven TiO_2_-Au Janus micromotors. *ACS Nano* **10**, 1, 839-844 (2016).
27. Ma, X. et al. Enzyme-powered hollow mesoporous Janus nanomotors. *Nano Lett.* **15**, 7043-7050 (2015).
28. Sanchez, S., Solovev, A. A., Mei, Y. F. & Schmidt, O. G. Dynamics of biocatalytic microengines mediated by variable friction control. *J. Am. Chem. Soc.* **132**, 13144-13145 (2010).
