# Supplementary material for: Bio-inspired nitric-oxide-driven nanomotor
Source: Nat Commun. 2019 Feb 27;10:966. doi: 10.1038/s41467-019-08670-8 (PMC6393443; doi:10.1038/s41467-019-08670-8)
Supplement: Supplementary file 4 — Description of Additional Supplementary Files [file 41467_2019_8670_MOESM4_ESM.pdf]

Description of Additional Supplementary Information Files

File Name: Supplementary Movie 1.

Description: The propulsion of HLAn nanomotors in H2O2 solution with multiple particles in one movie (20%).

File Name: Supplementary Movie 2.

Description: The propulsion of HLAn nanomotors in H2O2 solution (20%).

File Name: Supplementary Movie 3.

Description: The propulsion of HLA10 nanomotors under different H2O2 concentrations.

File Name: Supplementary Movie 4.

Description: The propulsion of HLA10 nanomotors under cell envioronment.

File Name: Supplementary Movie 5.

Description: 3D rendered Movie made from a stack of confocal images for cell uptake of HLA10 nanomotors by MCF-7.

File Name: Supplementary Movie 6.

Description: 3D rendered Movie made from a stack of confocal images for cell uptake of HLA10 nanomotors by HUVECs.

File Name: Supplementary Movie 7.

Description: The propulsion of different nanomotors in H2O2 solution (20%).

File Name: Supplementary Movie 8.

Description: 3D rendered Movie made from a stack of confocal images for cell uptake of HLA10 and HFLA10 nanomotors by MCF-7
